# Adjuvant Chemotherapy Use for Hormone Receptor–Positive, *ERBB2*-Negative Breast Cancer After RxPONDER Trial

**DOI:** 10.1001/jamanetworkopen.2025.49109

**Published:** 2025-12-01

**Authors:** Jincong Q. Freeman, Poornima Saha, Daniel S. Peiffer, Nan Chen, Sarah P. Shubeck, Sudha R. Yarlagadda, Rita Nanda, Dezheng Huo, Frederick M. Howard

**Affiliations:** Department of Public Health Sciences, The University of Chicago, Chicago, Illinois; Cancer Prevention and Control Research Program, UChicago Medicine Comprehensive Cancer Center, Chicago, Illinois; Center for Health and the Social Sciences, The University of Chicago, Chicago, Illinois; Division of Hematology and Oncology, Department of Medicine, Endeavor Health, Evanston, Illinois; Section of Hematology and Oncology, Department of Medicine, The University of Chicago, Chicago, Illinois; Section of Hematology and Oncology, Department of Medicine, The University of Chicago, Chicago, Illinois; Department of Surgery, The University of Chicago Medicine, Chicago, Illinois; Section of Hematology and Oncology, Department of Medicine, The University of Chicago, Chicago, Illinois; Section of Hematology and Oncology, Department of Medicine, The University of Chicago, Chicago, Illinois; Department of Public Health Sciences, The University of Chicago, Chicago, Illinois; Cancer Prevention and Control Research Program, UChicago Medicine Comprehensive Cancer Center, Chicago, Illinois; Section of Hematology and Oncology, Department of Medicine, The University of Chicago, Chicago, Illinois

## Abstract

**IMPORTANCE:**

Since 2018, the TAILORx and RxPONDER trials have demonstrated that the 21-gene recurrence score (RS) can be indicative of the benefit of adjuvant chemotherapy in hormone receptor (HR)–positive, *ERBB2* (formerly *HER2*)–negative breast cancer with 3 or fewer positive lymph nodes. However, its applicability to key subgroups with high risk for recurrence, including premenopausal women with positive lymph nodes and racial and ethnic minority individuals, remains unclear.

**OBJECTIVE:**

To assess the temporal patterns of and disparities in adjuvant chemotherapy use in early-stage HR-positive, *ERBB2*-negative breast cancer by age, genomic risk, and nodal involvement.

**DESIGN, SETTING, AND PARTICIPANTS:**

This retrospective cohort study analyzed clinical data from the 2010 to 2022 National Cancer Database. The cohort included women with stage I to III, HR-positive, *ERBB2*-negative breast cancer who had undergone a lumpectomy or mastectomy and were eligible for endocrine therapy. Patients were categorized into premenopausal (aged ≤50 years) or postmenopausal (aged >50 years) status. Nodal status (negative or positive) was pathologically confirmed. RS was classified per the TAILORx trial, with RS of 0 to 10 as low genomic risk, RS of 11 to 25 as intermediate genomic risk, and RS of 26 or higher as high genomic risk. Data were analyzed from January 20 to August 11, 2025.

**MAIN OUTCOMES AND MEASURES:**

Adjuvant systemic therapy, defined as receipt of either endocrine therapy alone or chemoendocrine therapy (chemotherapy plus endocrine therapy), after surgery (lumpectomy or mastectomy).

**RESULTS:**

A total of 504 937 women (mean [SD] age, 60.0 [10.7] years; 5.4% Hispanic, 4.3% non-Hispanic Asian or Pacific Islander, 8.1% non-Hispanic Black, 81.3% non-Hispanic White, and 0.9% other race or ethnicity) were included. Among premenopausal patients with node-negative tumors, adjuvant chemotherapy use decreased from 6.5% in 2010 to 0.9% in 2022 for those with low genomic risk and from 29.6% in 2010 to 11.1% in 2022 for those with intermediate genomic risk. However, among premenopausal patients with node-positive disease, chemotherapy use declined from 33.3% in 2010 to 12.7% in 2019 but increased to 25.7% in 2022 for the low genomic risk group. For the intermediate genomic risk group, chemotherapy use declined from 55.8% in 2010 to 38.1% in 2019 but increased to 48.9% in 2022. Among postmenopausal women, chemotherapy use for those with low to intermediate genomic risk continued to decrease from 2010 to 2022 in both node-negative and node-positive disease status. Black women with high genomic risk had lower odds of chemotherapy receipt than White women, regardless of menopausal or nodal status (adjusted odds ratio [AOR], 0.84; 95% CI, 0.78–0.90). Premenopausal Black women with low to intermediate genomic risk also had lower odds of chemotherapy receipt than White women (AOR, 0.85; 95% CI, 0.77–0.94), regardless of nodal status.

**CONCLUSIONS AND RELEVANCE:**

This retrospective cohort study found that adjuvant chemotherapy use almost doubled in premenopausal patients with node-positive tumors and with a low to intermediate genomic risk from 2019 to 2022 but decreased for patients with node-negative disease, coinciding with the publication of the TAILORx and RxPONDER trials. The findings highlight the variability in genomic assay use to facilitate adjuvant therapy recommendations for HR-positive, *ERBB2*-negative breast cancer.

## Introduction

In the US, breast cancer is the most commonly diagnosed female malignant neoplasm,^1^ with 70% of diagnoses being hormone receptor (HR) positive and *ERBB2* (formerly *HER2*) negative.^2^ While many women with early-stage HR-positive, *ERBB2*-negative breast cancer do well with adjuvant endocrine therapy alone, some patients benefit from the addition of adjuvant chemotherapy to further reduce the risk of recurrence and improve survival outcomes. Chemotherapy use is associated with increased risk of short-term and late-term toxic effects and substantial treatment costs.^3–6^ The 21-gene recurrence score (RS) has been extensively validated and is a common tool in clinical practice to guide the use of chemotherapy with endocrine therapy.^7,8^

Since 2018, the landmark TAILORx (Hormone Therapy With or Without Combination Chemotherapy in Treating Women Who Have Undergone Surgery for Node-Negative Breast Cancer; NCT00310180) and RxPONDER (Tamoxifen Citrate, Letrozole, Anastrozole, or Exemestane With or Without Chemotherapy in Treating Patients With Invasive RxPONDER Breast Cancer; NCT01272037) trials have influenced adjuvant chemotherapy practice patterns.^9–11^ In 2018, the TAILORx trial evaluated women with HR-positive, *ERBB2*-negative, node-negative breast cancer and found no chemotherapy benefit in women older than 50 years with low to intermediate genomic risk (RS ≤25). Among premenopausal women 50 years or younger, however, a modest benefit was noted among those with an RS of 21 to 25 or 16 to 20 with higher clinical risk.^9,10^ In 2020, the RxPONDER trial evaluated patients with HR-positive, *ERBB2*-negative breast cancer with up to 3 positive lymph nodes; no chemotherapy benefit was seen in patients with low to intermediate genomic risk (RS ≤25). Among premenopausal women with 1 to 3 nodes, the RxPONDER trial revealed up to a 5% absolute benefit of chemotherapy, regardless of genomic risk, potentially due to chemotherapy-induced ovarian failure rather than cytotoxic effects of chemotherapy.^11^ Premenopausal women with HR-positive, *ERBB2*-negative breast cancer are at higher risk for recurrence,^12^ but RS alone appears to be less reliable in this patient group.

Data are also conflicting regarding the ability of the 21-gene assay to estimate chemotherapy benefit across all racial and ethnic groups. Previous studies have demonstrated that the 21-gene RS may have less prognostic accuracy in Black women.^13–15^ Uncertainty exists regarding the applicability of RS in key high-risk subgroups of patients with early-stage HR-positive, *ERBB2*-negative breast cancer. Although younger women with node-positive disease and Black women are at higher risk of recurrence,^16^ the extent to which the 21-gene RS can identify chemotherapy benefit in these patient groups remains controversial.^17^ Current national patterns of adjuvant chemotherapy use in this patient population are also unclear. To address these knowledge gaps, we conducted this study with the aim to assess the temporal patterns of and disparities in adjuvant chemotherapy use in early-stage HR-positive, *ERBB2*-negative breast cancer by age, genomic risk, and nodal involvement.

## Methods

### Study Design and Data Source

This retrospective cohort study analyzed clinical data obtained from the 2010 to 2022 National Cancer Database (NCDB). The NCDB, a joint project of the Commission on Cancer (CoC) of the American College of Surgeons and the American Cancer Society,^18^ is a national clinical oncology registry that captures approximately 72% of new cancer diagnoses in the US from more than 1500 CoC-accredited cancer centers each year.^19,20^ The University of Chicago institutional review board deemed this study exempt from ethics review and waived the informed consent requirement because the NCDB contains deidentified data of hospitals, health care practitioners, and patients. We followed the Strengthening the Reporting of Observational Studies in Epidemiology (STROBE) reporting guideline.^21^

### Patient Cohorts and Measures

We identified breast cancer diagnoses by reviewing the *International Classification of Diseases for Oncology, Third Revision* (*ICD-O-3*) codes C50.0 to C50.9 (except for *ICD-O-3* codes 9727, 9732, 9741–9742, 9749, 9762–9809, 9832, 9840–9931, 9945–9946, 9950–9968, and 9975–9993) for breast as the primary site recorded in the NCDB.^18^ Sample selection was illustrated in eFigure 1 in Supplement 1. We included patients who were 18 years or older; female sex at birth; diagnosed with stage I to III, HR-positive and *ERBB2*-negative breast cancer and had undergone a lumpectomy or mastectomy; and eligible for endocrine therapy between January 1, 2010, and December 31, 2022. Patients without information on nodal status and/or the 21-gene RS were excluded. In our sensitivity analysis, the sample was limited to the subgroup of patients who were diagnosed between January 1, 2018, and December 31, 2022, following the presentation and publication of the TAILORx trial results.

The primary outcome of interest was adjuvant systemic therapy, which was defined as receipt of either endocrine therapy alone or chemoendocrine therapy (chemotherapy in addition to endocrine therapy) after surgery (lumpectomy or mastectomy). Based on age at diagnosis, patients were stratified into likely premenopausal (aged ≤50 years) or postmenopausal (aged >50 years) cohorts. Nodal status, determined as negative (pN0) or positive (pN1+), was pathologically confirmed for lymph node involvement. The 21-gene RS was classified as low genomic risk (RS 0–10), intermediate genomic risk (RS 11–25), or high genomic risk (RS ≥26) per the TAILORx trial cutoffs.^9^ We further classified the intermediate risk category into 3 granular RS groups: 11 to 15, 16 to 20, and 21 to 25.

Furthermore, we constructed 3 specific patient cohorts according to the TAILORx and RxPONDER trials and the National Comprehensive Cancer Network (NCCN) Guidelines for Breast Cancer.^9–11,22^ Cohort 1 included patients with high genomic risk (RS ≥26), regardless of menopausal and nodal status. Cohort 2 included premenopausal women with negative nodes and intermediate genomic risk (RS <16) as well as postmenopausal women with negative or positive nodes and low to intermediate genomic risk (RS 0–25). Cohort 3 included premenopausal women with negative nodes and intermediate genomic risk (RS 16–25) or positive nodes with low to intermediate genomic risk (RS 0–25).

### Covariates

Per the NCDB, race and ethnicity were self-reported. Patients reported their primary race and Spanish or Hispanic ethnicity separately. We categorized race and ethnicity into 5 groups: Hispanic, non-Hispanic Asian or Pacific Islander (hereafter, Asian or Pacific Islander), non-Hispanic Black (hereafter, Black), non-Hispanic White (hereafter, White), and Other (American Indian, Alaska Native, other, or unknown). Other is a racial and ethnic group listed in the NCDB, representing patients categorized as other by local cancer registries. The NCDB does not define race and ethnicity categorized as other. For temporal patterns, we included year of initial breast cancer diagnosis, spanning 2010 through 2022.

Additional covariates included primary payer (Medicaid, Medicare, other governmental, private or managed care, or uninsured), area-level educational attainment, median household income quartile, rural-urban residence, facility type or cancer program, Charlson-Deyo Comorbidity Index score, histologic type (ductal, ductal and lobular, lobular, or other), American Joint Committee on Cancer pathologic T stage, and tumor grade. Area-level educational attainment was classified using quartiles of percentage without a high school diploma, which was measured by matching patients’ residential zip codes recorded at the time of diagnosis to files derived from the 2020 American Community Survey data. Median household income quartile was based on the 2020 American Community Survey data, spanning 2016 through 2020 and adjusted for 2016 inflation.^23^ Rural-urban residence was measured by matching the state and county Federal Information Processing Standards code of the patient recorded at the time of diagnosis to 2013 files published by the US Department of Agriculture Economic Research Service.^18^ Facility type was defined by CoC accreditation based on program structure, care and services provided, and number of annual cases reported and was classified into academic or research, community, comprehensive community, or integrated network.

### Statistical Analysis

We first used summary statistics to describe patient characteristics by expected menopausal status (aged ≤50 years [premenopausal] or >50 years [postmenopausal]) and by the 21-gene RS category (low, intermediate, or high genomic risk). Mean (SD) and median (IQR) were calculated for continuous variables and compared using unpaired, 2-tailed *t* tests or Wilcoxon rank sum tests. For categorical data, we tabulated frequencies (%) and compared the distributions using Pearson χ^2^ tests. To evaluate the temporal patterns of adjuvant chemotherapy use from 2010 to 2022, we computed the percentages of chemotherapy use stratified by nodal status across RS categories in both premenopausal and postmenopausal cohorts. To assess factors associated with adjuvant chemotherapy use and racial and ethnic differences, we fit multivariable logistic regression models across the 3 treatment cohorts separately. All regression models were controlled for age group, nodal involvement, the 21-gene RS category, pathologic T stage, histologic type, tumor grade, Charlson-Deyo Comorbidity Index score, year of initial diagnosis, area-level educational attainment quartile, median household income quartile, primary payer, and facility type. We used the same regression approaches in the sensitivity analysis restricted to the 2018 to 2022 subgroup. Adjusted odds ratios (AORs) and 95% CIs were calculated. A 2-sided *P* < .05 was considered statistically significant. All statistical analyses were performed from January 20 to August 11, 2025, using Stata, version 18.0 (StataCorp LLC).

## Results

### Patient Characteristics

A total of 504 937 women (mean [SD] age, 60.0 [10.7] years) were included. Of these patients, 4.3% identified as Asian or Pacific Islander, 8.1% as Black, 5.4% as Hispanic, 81.3% as White, and 0.9% as other race or ethnicity; 21.2% were premenopausal and 78.8% were postmenopausal; and 81.4% had node-negative and 18.6% had node-positive tumors (Table 1). Among these patients, 61.0% had an intermediate genomic risk, followed by 24.6% with a low genomic risk and 14.4% with a high genomic risk. Additionally, 56.7% of patients were privately insured, and 35.6% were covered by Medicare; 46.0% had a median household income of $74 063 or higher, and 29.0% lived in a neighborhood with less than 5.0% of residents having no high school diploma. Sociodemographic and clinicopathologic characteristics were significantly different between premenopausal and postmenopausal patients (Table 1) and across RS categories (eTable 1 in Supplement 1).

### Patterns of Adjuvant Chemotherapy Use

**Figure 1.**
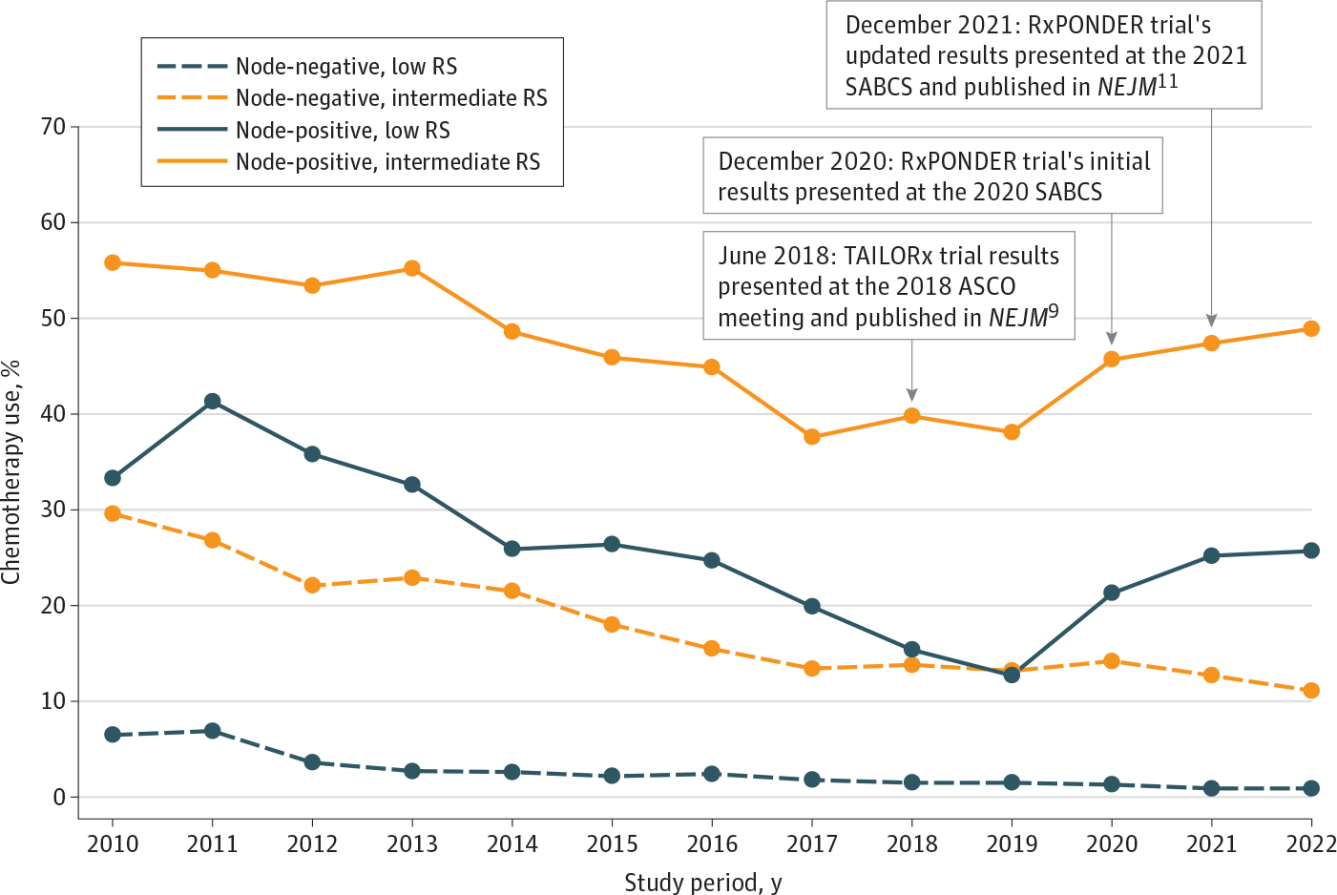
Patterns of Adjuvant Chemotherapy Use Among Premenopausal Women With Stage I to III Hormone Receptor–Positive, *ERBB2*-Negative Breast Cancer Across Genomic Risk Categories, Stratified by Nodal Status ASCO indicates American Society of Clinical Oncology; *NEJM*, *New England Journal of Medicine*; RS, recurrence score; SABCS, San Antonio Breast Cancer Symposium. Low genomic risk: RS 0 to 10; intermediate genomic risk: RS 11 to 25; high genomic risk: RS ≥26.

In premenopausal patients with node-negative disease, adjuvant chemotherapy use decreased from 6.5% in 2010 to 0.9% in 2022 for those with low genomic risk and from 29.6% in 2010 to 11.1% in 2022 for those with intermediate genomic risk (Figure 1). Similar decreasing patterns of adjuvant chemotherapy use were observed among patients with node-negative disease with an RS of 11 to 15, 16 to 20, or 21 to 25 (eFigure 2 in Supplement 1). However, in premenopausal women with positive nodes, adjuvant chemotherapy use declined from 33.3% in 2010 to 12.7% in 2019 but increased to 25.7% in 2022 for the low genomic risk group and declined from 55.8% in 2010 to 38.1% in 2019 but increased to 48.9% in 2022 for the intermediate genomic risk group (Figure 1). We also observed similar patterns of increased adjuvant chemotherapy use from 2019 to 2022 in patients with positive nodes and an RS of 11 to 15, 16 to 20, or 21 to 25 (eFigure 3 in Supplement 1).

**Figure 2.**
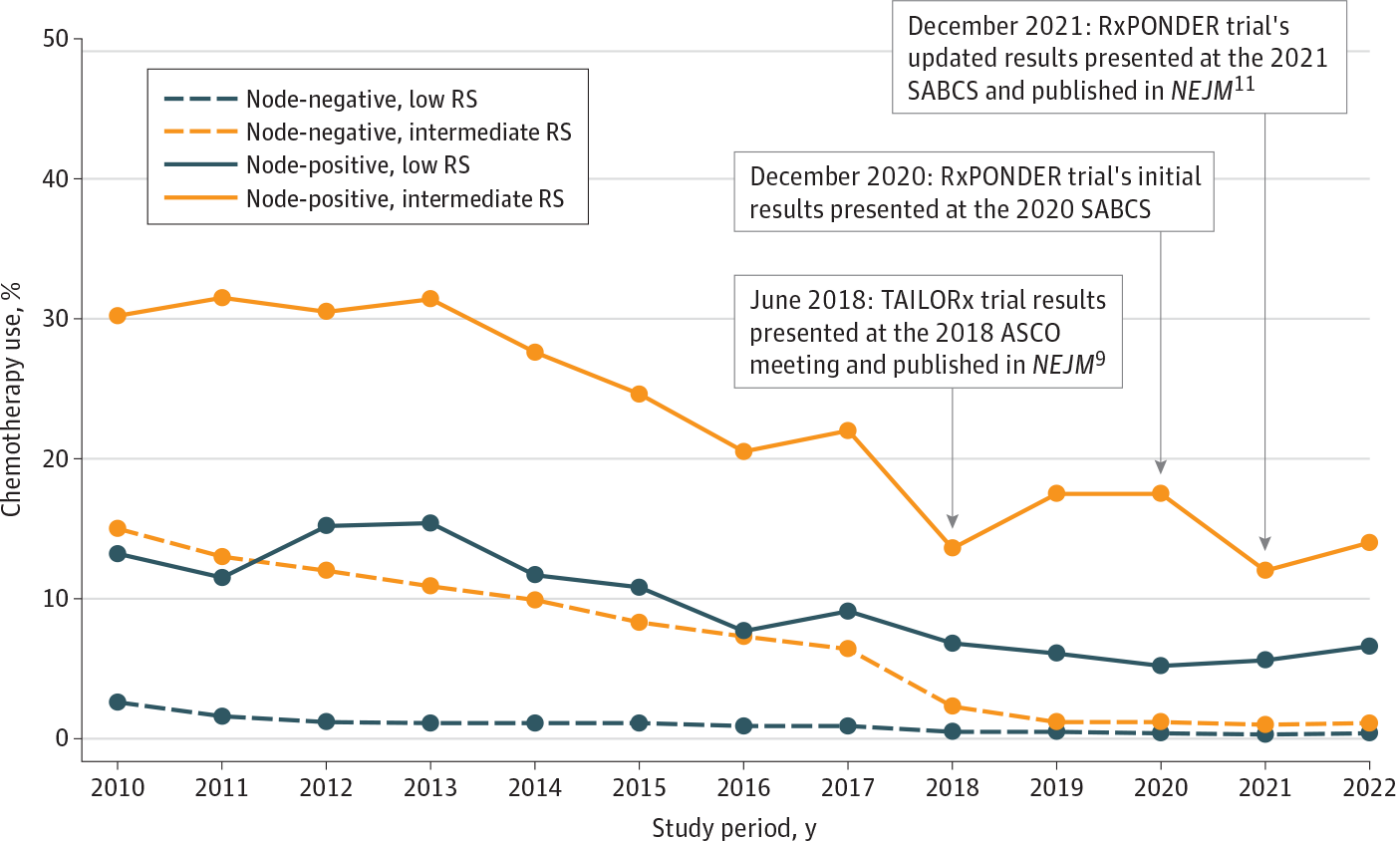
Patterns of Adjuvant Chemotherapy Use Among Postmenopausal Women With Stage I to III Hormone Receptor–Positive, *ERBB2*-Negative Breast Cancer Across Genomic Risk Categories, Stratified by Nodal Status ASCO indicates American Society of Clinical Oncology; *NEJM*, *New England Journal of Medicine*; RS, recurrence score; SABCS, San Antonio Breast Cancer Symposium. Low genomic risk: RS 0 to 10; intermediate genomic risk: RS 11 to 25; high genomic risk: RS ≤26.

Among postmenopausal women with node-negative tumors, adjuvant chemotherapy use decreased from 2.6% in 2010 to 0.4% in 2022 for the low genomic risk group and decreased from 15.0% in 2010 to 1.1% in 2022 for the intermediate genomic risk group. Similarly, among postmenopausal women with node-positive tumors, adjuvant chemotherapy use declined from 13.2% in 2010 to 6.6% in 2022 for those with low genomic risk and declined from 30.2% in 2010 to 14.0% in 2022 for those with intermediate genomic risk (Figure 2). These decreasing patterns of adjuvant chemotherapy use were also observed among patients with an RS of 11 to 15, 16 to 20, or 21 to 25 in both node-negative and node-positive cohorts. Regardless of nodal and menopausal status, adjuvant chemotherapy use for the high genomic risk group was constant over the study period (eFigures 4–5 in Supplement 1).

### Clinical and Demographic Patterns of Adjuvant Chemotherapy Use

The distribution of sociodemographic and clinicopathologic factors by treatment modality (chemoendocrine therapy [n = 94 651, 18.8%] vs endocrine therapy alone [n = 408 930, 81.2%]) is presented in eTable 2 in Supplement 1. Compared with patients who received endocrine therapy alone, those who received chemoendocrine therapy were younger (mean [SD] age, 60.9 [10.4] vs 56.0 [11.1] years; *P* < .001) and more likely to be Black (7.7% vs 10.0%; *P* < .001) or Hispanic (5.2% vs 6.2%; *P* < .001), privately insured (54.5% vs 66.2%; *P* < .001), and diagnosed with stage II (16.0% vs 34.7%; *P* < .001) or stage III (0.5% vs 3.2%; *P* < .001) disease. In premenopausal women with node-positive tumors and intermediate genomic risk, 5957 patients (46.1%) received chemoendocrine therapy and 6957 patients (53.9%) received endocrine therapy alone. Furthermore, adjuvant chemotherapy use was found to be significantly different between RS categories, with women with higher RS more likely to have received chemoendocrine therapy than endocrine therapy alone in the overall population, as well as stratified by nodal status (eTable 3 in Supplement 1).

In cohort 1 (high genomic risk; n = 72 555), Black women (AOR, 0.84; 95% CI, 0.78–0.90) had lower odds of adjuvant chemotherapy use than White women (Table 2). The odds of adjuvant chemotherapy use were higher in patients with node-positive than node-negative tumors (AOR, 1.35; 95% CI, 1.27–1.44). Compared with patients with private insurance, those without insurance (AOR, 0.75; 95% CI, 0.61–0.93) and Medicaid (AOR, 0.79; 95% CI, 0.71–0.88) or Medicare (AOR, 0.73; 95% CI, 0.69–0.78) beneficiaries had lower odds of having received adjuvant chemotherapy.

In cohort 2 (low genomic risk, and chemotherapy was not recommended; n = 367 547), Hispanic women had greater odds of adjuvant chemotherapy use than White women (AOR, 1.13; 95% CI, 1.04–1.22) (Table 2). The odds of adjuvant chemotherapy use were higher among patients with positive nodes than those with negative nodes (AOR, 5.21; 95% CI, 5.01–5.41). Compared with academic or research programs, community programs were more likely to use adjuvant chemotherapy (AOR, 1.13; 95% CI, 1.05–1.22), while comprehensive community (AOR, 0.95; 95% CI, 0.91–0.99) or integrated network (AOR, 0.94; 95% CI, 0.89–0.99) programs were less likely to do so.

In cohort 3 (low to intermediate genomic risk, and chemotherapy benefit was unclear; n = 48 553), Black patients had lower odds of having received adjuvant chemotherapy than White patients (AOR, 0.85; 95% CI, 0.77–0.94) (Table 2). The odds of adjuvant chemotherapy use were lower in Medicare beneficiaries than in patients with private insurance (AOR, 0.55; 95% CI, 0.46–0.67).

Consistent across these 3 cohorts, older age groups were associated with lower odds of adjuvant chemotherapy use. Higher 21-gene RS, stage pT2 to pT4 tumors, and tumor grades 2 to 3 were associated with greater odds of adjuvant chemotherapy use. Similar results were observed in the sensitivity analysis (eTable 4 in Supplement 1).

## Discussion

Use of the 21-gene RS has been associated with a significant decline in adjuvant chemotherapy use in early-stage HR-positive, *ERBB2*-negative breast cancer in the US, with many patients appropriately spared the added toxic effects of cytotoxic chemotherapy when found to have a genomically low risk of breast cancer recurrence.^24^ Clinical controversy remains,^23^ however, regarding the use of genomic assays to guide treatment recommendation across all populations, such as for younger patients and racial and ethnic minority patients. In this retrospective analysis of early-stage HR-positive, *ERBB2*-negative breast cancer, we observed differences in adjuvant chemotherapy use among premenopausal women by genomic risk and nodal involvement, highlighting the variability in the use of genomic assays to facilitate adjuvant systemic therapy recommendations.

Results of this study show a substantial shift in treatment paradigms for premenopausal women with node-positive tumors in the post-RxPONDER trial era. There was an almost doubling (12.7% to 25.7%) of adjuvant chemotherapy use for premenopausal women with node-positive disease and an RS lower than 26 from 2019 to 2022, likely associated with the recurrence benefit reported with adjuvant chemotherapy for this group in the RxPONDER trial. It is unclear whether some of the potential benefits of adjuvant chemotherapy in this premenopausal population are due to chemotherapy-induced menopause rather than the direct cytotoxic effect of chemotherapy. Our findings reflect the clinical equipoise in this patient population, given that, among premenopausal women with positive nodes and an intermediate genomic risk, 46.1% received chemoendocrine therapy and 53.9% were treated with endocrine therapy alone. The NCCN guidelines support ovarian suppression alone or with chemotherapy in this patient population.^22^ The OFSET trial (Evaluating the Addition of Adjuvant Chemotherapy to Ovarian Function Suppression Plus Endocrine Therapy in Premenopausal Patients With pN0–1, ER-Positive/HER2-Negative Breast Cancer and an Oncotype Recurrence Score Less Than or Equal to 25; NCT05879926),^25^ which directly compares endocrine therapy against ovarian suppression with or without chemotherapy in premenopausal women with low to intermediate genomic risk, may be able to clarify the best treatment approach in this population. Additionally, recent studies applying artificial intelligence approaches to clinical parameters^26,27^ or the combination of clinical parameters and histopathology^28,29^ have identified patients at low risk of recurrence who may benefit from chemotherapy-sparing approaches despite classically high-risk disease.

Consistent with subsequent analyses of the TAILORx trial (where premenopausal women with high clinicopathologic risk benefit more from chemotherapy),^10^ higher RS, tumor grades 2 to 3, and pT2 to pT4 tumors were associated with greater odds of chemotherapy use. We also found socioeconomic factors associated with less chemotherapy receipt and potential for undertreatment, including older age, Black race, Medicaid and Medicare coverage or lack of insurance, and lower median household income. In cohort 3, Black women were less likely to have received adjuvant chemotherapy as compared with White women. Similar racial differences in chemotherapy use were observed in cohort 1 patients with high genomic risk. However, Hispanic women in cohort 2 were more likely than White women to have received chemotherapy, suggesting potential overtreatment in this group of patients with low genomic risk. Multiple factors may contribute to these findings, including social determinants of health, clinician bias, and/or cultural preferences regarding chemotherapy use.^30–33^ For example, a prior study found that Black patients had a 3% greater likelihood of declining recommended neoadjuvant or adjuvant chemotherapy compared with White patients, while Hispanic patients were 22% less likely to do so.^30^ Future studies are needed to explore how these factors affect adjuvant chemotherapy use in women with early-stage HR-positive, *ERBB2*-negative breast cancer, particularly in younger women.

Additionally, in this study, women with high genomic risk without insurance or with Medicaid or Medicare coverage were less likely than those with private insurance to have received adjuvant chemotherapy, regardless of menopausal or nodal status. Facility type may also play a role; we found that in cohort 2, comprising women who should not receive chemotherapy per the NCCN guidelines, both comprehensive community and integrated network cancer programs were less likely to prescribe adjuvant chemotherapy, indicating adherence to current clinical practice guidelines, whereas community programs were more likely to recommend adjuvant chemotherapy. Future research is required to elucidate these patterns’ impact and contribution to clinical practice. It is imperative that we continue to personalize therapy to ensure that patients at high genomic risk receive appropriate systemic therapy and that patients at low genomic risk can be appropriately selected to de-escalate and spare them from the additional toxic effect while reducing health care cost.

### Strengths and Limitations

To our knowledge, this study was the first to assess the patterns and disparities in adjuvant systemic therapy for early-stage HR-positive, *ERBB2*-negative breast cancer in the post-RxPONDER trial era. Strengths of our analysis include the use of a large and diverse patient population in the US. It is worth noting that the COVID-19 pandemic may have influenced chemotherapy recommendations. If the pandemic was a factor, we would expect a decreasing pattern of chemotherapy use from 2020 to 2021 and potentially increasing in 2022. However, we did not observe such a pattern and believe that the pandemic’s implication for adjuvant chemotherapy use was subtle.

This study has several limitations and potential unmeasured confounding variables. As in other retrospective studies, in this study menopause status could not be defined per standard NCCN definition. Age was used as a proxy for menopause status, and potential misclassification of menopause status is possible. Underreporting and reporting errors of therapy use are also possible given the nature of the NCDB. Genomic assay testing was selected by individual physicians and may have been biased due to patient selection and variability in clinical practice patterns. Additionally, the specific chemotherapy and endocrine therapy agents administered are not listed in the NCDB. Further study is needed to explore the disparities in the use of ovarian suppression among patients who refuse chemotherapy or in the appropriate use of anthracycline-sparing regimens in patients with low genomic risk.^34,35^ Due to the small number of patients self-reporting as American Indian, Alaska Native, other, or unknown, we categorized these identities as Other and were unable to examine group-specific differences. Although the NCDB is a national registry, this study is not a population-based study. Therefore, the sample may not represent all patients with early-stage HR-positive, *ERBB2*-negative breast cancer in the US, limiting the generalizability of the findings.

## Conclusions

In this large cohort study of patients with early-stage HR-positive, *ERBB2*-negative breast cancer, adjuvant chemotherapy use almost doubled in premenopausal women with node-positive tumors and a low to intermediate genomic risk but decreased for patients with node-negative tumors from 2019 to 2022, coinciding with the publication of the TAILORx and RxPONDER trials. These findings demonstrate the variability of genomic assay use to facilitate adjuvant therapy recommendations. The results of ongoing trials (eg, OFSET) will help to determine whether ovarian suppression alone is sufficient in this patient population.

## Supplementary Material

Supplement 1 **eFigure 1.** CONSORT Flow Diagram for Cohort Selection of Women Diagnosed With Stage I-III, HR-Positive/HER2-Negative Breast Cancer From the National Cancer Database
**eTable 1.** Sociodemographic and Clinicopathologic Characteristics of Women With Stage I-III, HR-Positive/HER2Negative Breast Cancer, by Genomic Risk
**eFigure 2.** Trends in Chemotherapy Use Among Premenopausal (Ages ≤50) Women With Stage I-III, HR-Positive/HER2-Negative, Node-Negative Breast Cancer Across Genomic Risk Categories
**eFigure 3.** Trends in Chemotherapy Use Among Premenopausal (Ages ≤50) Women With Stage I-III, HR-Positive/HER2-Negative, Node-Positive Breast Cancer Across Genomic Risk Categories
**eFigure 4.** Trends in Chemotherapy Use Among Postmenopausal (Ages >50) Women With Stage I-III, HR-Positive/HER2-Negative, Node-Negative Breast Cancer Across Genomic Risk Categories
**eFigure 5.** Trends in Chemotherapy Use Among Postmenopausal (Ages >50) Women With Stage I-III, HR-Positive/HER2-Negative, Node-Positive Breast Cancer Across Genomic Risk Categories
**eTable 2.** Sociodemographic and Clinicopathologic Characteristics of Women With Stage I-III, HR-Positive/HER2-Negative Breast Cancer, by Treatment Modality
**eTable 3.** Percentages of Adjuvant Chemotherapy Use in Women With Stage I-III, HR− Positive/HER2-Negative Breast Cancer Across Genomic Risk Categories, Overall and Stratified by Menopausal and Nodal Status
**eTable 4.** Factors Associated with Chemoendocrine Therapy Use (vs Endocrine Therapy Alone) in Stage I-III, Hormone Receptor-Positive/HER2-Negative Breast Cancer Across Patient Cohorts, 2018–2022

Supplement 2 SUPPLEMENT 2.Data Sharing Statement

## Figures and Tables

**Table 1.** Patient Sociodemographic and Clinicopathologic Characteristics

Characteristic	Patients, No. (%)			*P* value^a^
	Overall (n = 504 937)	Premenopausal (n = 107 206 [21.2])	Postmenopausal (n = 397 731 [78.8])	
Age at diagnosis, y				
Mean (SD)	60.0 (10.7)	44.6 (4.8)	64.2 (7.7)	<.001
Median (IQR)	61.0 (52.0–68.0)	46.0 (42.0–48.0)	64.0 (58.0–70.0)	<.001
Race and ethnicity^b^				
Hispanic	27 089 (5.4)	8651 (8.1)	18 438 (4.7)	<.001
Non-Hispanic Asian or Pacific Islander	21 772 (4.3)	7704 (7.2)	14068 (3.6)	
Non-Hispanic Black	40 603 (8.1)	9260 (8.7)	31 343 (7.9)	
Non-Hispanic White	407 209 (81.3)	79 426 (74.7)	327 783 (83.0)	
Other^c^	4477 (0.9)	1232 (1.2)	3245 (0.8)	
% Of neighborhood residents without a high school diploma, quartiles^d^				
≥15.3	63 509 (14.8)	13 321 (14.5)	50 188 (14.9)	<.001
9.1–15.2	107 061 (25.0)	21 232 (23.1)	85 829 (25.5)	
5.0–9.0	133 183 (31.1)	28 005 (30.5)	105 178 (31.3)	
<5.0	124 049 (29.0)	29 200 (31.8)	94 849 (28.2)	
Median household income quartiles, $^e^				
<46 277	49 387 (11.6)	9005 (9.8)	40 382 (12.0)	<.001
46 227–57 856	80 246 (18.8)	14892 (16.3)	65 354 (19.5)	
57857–74062	100 739 (23.6)	20 239 (22.1)	80 500 (24.0)	
≥74 063	196 451 (46.0)	47 484 (51.8)	148 967 (44.4)	
Primary payer at diagnosis				
Uninsured	5472 (1.1)	1930 (1.8)	3542 (0.9)	<.001
Private	283 558 (56.7)	90 685 (85.4)	192 873 (48.9)	
Medicaid	27 438 (5.5)	9604 (9.0)	17 834 (4.5)	
Medicare	178 390 (35.6)	2437 (2.3)	175 953 (44.6)	
Other governmental	5584 (1.1)	1492 (1.4)	4092 (1.0)	
Rural-urban residence^f^				
Metropolitan	412 585 (84.6)	89 371 (86.7)	323 214 (84.0)	<.001
Urban	65 562 (13.4)	12 059 (11.7)	53 503 (13.9)	
Rural	9653 (2.0)	1710 (1.7)	7943 (2.1)	
Facility type or cancer program				
Community	32 216 (6.6)	4823 (5.2)	27 393 (6.9)	<.001
Comprehensive community	198 763 (40.5)	34 590 (37.2)	164 173 (41.3)	
Academic or research	156 064 (31.8)	33 819 (36.4)	122 245 (30.7)	
Integrated network	103 634 (21.1)	19 714 (21.2)	83 920 (21.1)	
Charlson-Deyo Comorbidity Index				
0	420 943 (83.4)	97 829 (91.3)	323 114 (81.2)	<.001
1	63 117 (12.5)	8059 (7.5)	55 058 (13.8)	
≥2	20 877 (4.1)	1318 (1.2)	19 559 (4.9)	
AJCC stage group				
I	401 195 (79.5)	84 928 (79.2)	316 267 (79.5)	.02
II	98 485 (19.5)	21 205 (19.8)	77 280 (19.4)	
III	5257 (1.0)	1073 (1.0)	4184 (1.1)	
Histologic type				
Ductal	387 374 (76.7)	86 343 (80.5)	301 031 (75.7)	<.001
Lobular	70 829 (14.0)	11 397 (10.6)	59 432 (14.9)	
Ductal and lobular	31 500 (6.2)	6329 (5.9)	25 171 (6.3)	
Other^g^	15234 (3.0)	3137 (2.9)	12097 (3.0)	
Progesterone receptor status				
Negative	47 723 (9.5)	4357 (4.1)	43 366 (10.9)	<.001
Positive	456 858 (90.5)	102 786 (95.9)	354 072 (89.1)	
AJCC pathologic T stage				
pT1	360 359 (72.7)	74 860 (71.3)	285 499 (73.0)	<.001
pT2	124 966 (25.2)	27 805 (26.5)	97 161 (24.8)	
pT3	10 018 (2.0)	2268 (2.2)	7750 (2.0)	
pT4	661 (0.1)	63 (0.1)	598 (0.2)	
AJCC pathologic nodal status				
pN0	395 614 (81.4)	85 034 (81.3)	310 580 (81.4)	.51
pN1	86 929 (17.9)	18 776 (17.9)	68 153 (17.9)	
pN2	2832 (0.6)	635 (0.6)	2197 (0.6)	
pN3	809 (0.2)	166 (0.2)	643 (0.2)	
Negative (pN0)	395 614 (81.4)	85 034 (81.3)	310 580 (81.4)	.42
Positive (pN1+)	90 570 (18.6)	19 577 (18.7)	70 993 (18.6)	
Tumor grade				
1	149 690 (31.2)	31 290 (30.8)	118 400 (31.3)	<.001
2	268 719 (56.0)	55 915 (55.0)	212 804 (56.2)	
3	61 730 (12.9)	14 545 (14.3)	47 185 (12.5)	
21-Gene RS				
Low genomic risk (RS 0–10)	124 312 (24.6)	21 580 (20.1)	102 732 (25.8)	<.001
Intermediate genomic risk (RS 11–25)	308 070 (61.0)	70 417 (65.7)	237 653 (59.8)	
High genomic risk (RS ≥26)	72 555 (14.4)	15 209 (14.2)	57 346 (14.4)	

Abbreviations: AJCC, American Joint Committee on Cancer; RS, recurrence score.

a *P* values were computed using unpaired, 2-tailed *t* tests; Wilcoxon rank sum tests; or Pearson χ^2^ tests.

b Self-reported by patients and obtained from the 2010–2022 National Cancer Database.

c Other included American Indian, Alaska Native, other (not defined), or unknown.

d Defined as educational attainment for residence areas and measured by matching the zip code of the patient recorded at the time of diagnosis to files derived from the 2020 American Community Survey data.

e Based on the 2020 American Community Survey data, spanning 2016 through 2020 and adjusted for 2016 inflation.

f Measured by matching the state and county Federal Information Processing Standards code of the patient recorded at the time of diagnosis to 2013 files published by the US Department of Agriculture Economic Research Service.

g Other histologic type included inflammatory, medullary, metaplastic, mucinous, or other not otherwise specified.

**Table 2.** Factors Associated With Use of Chemoendocrine Therapy vs Endocrine Therapy Alone Across Patient Cohorts

Characteristic	AOR (95% CI)		
	Cohort 1: patients with RS ≥26, regardless of menopausal and nodal status^a^	Cohort 2: premenopausal patients with pN0 and RS <16; postmenopausal patients with pN0 or pN1+ and RS ≤25^a^	Cohort 3: premenopausal patients with pN0 and RS 16–25 or pN1+ and RS 0–25^a^
No. of patients	72 555	367 547	48 553
Race and ethnicity			
Hispanic	1.05 (0.94–1.17)	1.13 (1.04–1.22)	1.07 (0.97–1.19)
Non-Hispanic Asian or Pacific Islander	1.00 (0.89–1.11)	0.96 (0.87–1.06)	0.97 (0.88–1.08)
Non-Hispanic Black	0.84 (0.78–0.90)	0.96 (0.90–1.03)	0.85 (0.77–0.94)
Non-Hispanic White	1 [Reference]	1 [Reference]	1 [Reference]
Other^b^	0.79 (0.62–0.99)	0.83 (0.67–1.01)	1.00 (0.78–1.29)
Age group, y			
≤40	1 [Reference]	1 [Reference]	1 [Reference]
41–50	0.76 (0.57–1.03)	0.56 (0.43–0.73)	0.67 (0.60–0.75)
51–60	0.50 (0.37–0.67)	0.54 (0.42–0.71)	NA
61–70	0.34 (0.25–0.45)	0.33 (0.26–0.43)	NA
≥71	0.13 (0.10–0.18)	0.15 (0.11–0.19)	NA
AJCC pathologic nodal status			
Negative (pN0)	1 [Reference]	1 [Reference]	1 [Reference]
Positive (pN1+)	1.35 (1.27–1.44)	5.21 (5.01–5.41)	5.11 (4.78–5.46)
21-gene RS	1.03 (1.03–1.04)	1.21 (1.21–1.22)	1.24 (1.23–1.25)
AJCC pathologic T stage			
pT1	1 [Reference]	1 [Reference]	1 [Reference]
pT2	1.20 (1.15–1.26)	1.70 (1.63–1.77)	1.68 (1.58–1.78)
pT3	1.12 (0.95–1.32)	5.11 (4.66–5.61)	5.05 (4.32–5.91)
pT4	1.30 (0.76–2.20)	5.08 (3.64–7.10)	3.19 (1.24–8.20)
Histologic type			
Ductal	1 [Reference]	1 [Reference]	1 [Reference]
Lobular	0.87 (0.81–0.94)	0.93 (0.89–0.98)	0.87 (0.80–0.95)
Ductal and lobular	0.83 (0.75–0.92)	0.97 (0.91–1.04)	0.85 (0.77–0.95)
Other^c^	1.11 (0.94–1.30)	0.87 (0.77–0.99)	0.82 (0.68–0.98)
Tumor grade			
1	1 [Reference]	1 [Reference]	1 [Reference]
2	1.57 (1.47–1.69)	1.44 (1.37–1.50)	1.52 (1.43–1.62)
3	2.38 (2.21–2.56)	2.60 (2.45–2.76)	2.51 (2.29–2.75)
Charlson-Deyo Comorbidity Index			
0	1 [Reference]	1 [Reference]	1 [Reference]
1	1.25 (1.14–1.38)	1.12 (1.01–1.24)	0.91 (0.72–1.16)
≥2	1.26 (1.13–1.41)	1.12 (1.00–1.25)	1.03 (0.80–1.34)
Years of initial diagnosis (2010 to 2022)	1.04 (1.04–1.05)	0.82 (0.81–0.82)	0.92 (0.91–0.93)
% Of neighborhood residents without a high school diploma, quartile^d^			
≥15.3	1 [Reference]	1 [Reference]	1 [Reference]
9.1–15.2	1.02 (0.95–1.10)	0.96 (0.90–1.02)	0.89 (0.81–0.98)
5.0–9.0	1.06 (0.99–1.15)	0.97 (0.91–1.04)	0.93 (0.84–1.02)
<5.0	1.10 (1.01–1.20)	0.94 (0.88–1.01)	0.89 (0.80–0.99)
Median household income quartile, $^e^			
<46 277	0.92 (0.85–1.01)	0.94 (0.88–1.01)	0.93 (0.83–1.04)
46 227–57 856	0.99 (0.93–1.06)	0.97 (0.92–1.02)	0.93 (0.86–1.02)
57 857–74 062	0.94 (0.88–1.00)	0.94 (0.89–0.99)	0.90 (0.83–0.97)
≥74 063	1 [Reference]	1 [Reference]	1 [Reference]
Primary payer at diagnosis			
Uninsured	0.75 (0.61–0.93)	0.79 (0.67–0.94)	1.00 (0.82–1.21)
Private	1 [Reference]	1 [Reference]	1 [Reference]
Medicaid	0.79 (0.71–0.88)	0.98 (0.91–1.06)	0.94 (0.86–1.04)
Medicare	0.73 (0.69–0.78)	0.80 (0.76–0.84)	0.55 (0.46–0.67)
Other governmental	1.11 (0.89–1.39)	0.95 (0.81–1.12)	0.93 (0.74–1.17)
Facility type or cancer program			
Community	0.98 (0.89–1.07)	1.13 (1.05–1.22)	1.02 (0.90–1.15)
Comprehensive community	0.97 (0.92–1.02)	0.95 (0.91–0.99)	0.90 (0.85–0.96)
Academic or research	1 [Reference]	1 [Reference]	1 [Reference]
Integrated network	1.08 (1.02–1.15)	0.94 (0.89–0.99)	0.95 (0.89–1.03)

Abbreviations: AJCC, American Joint Committee on Cancer; AOR, adjusted odds ratio; NA, not applicable; RS, recurrence score.

a Low genomic risk: RS 0–10; intermediate genomic risk: RS 11–25; high genomic risk: RS ≥26.

b Other includes American Indian, Alaska Native, Other, or unknown races and ethnicities.

c Other histologic type included inflammatory, medullary, metaplastic, mucinous, or other not otherwise specified.

d Defined as educational attainment for residence areas and measured by matching the zip code of the patient recorded at the time of diagnosis to files derived from the 2020 American Community Survey data.

e Based on the 2020 American Community Survey data, spanning 2016 through 2020 and adjusted for 2016 inflation.

## Data Availability

See Supplement 2.

## Key Points

### Question

What are the patterns of adjuvant chemotherapy use for early-stage hormone receptor (HR)–positive, *ERBB2-*negative breast cancer by genomic risk and nodal status?

### Findings

In this cohort study of 504 937 women, adjuvant chemotherapy use nearly doubled in premenopausal patients with node-positive tumors and low or intermediate 21-gene recurrence score from 2019 to 2022 but decreased for women with node-negative disease.

### Meaning

The findings highlight the variability in genomic assay use to inform adjuvant systemic therapy recommendations in HR-positive, *ERBB2*-negative breast cancer.
