# Supplementary material for: Adjuvant Chemotherapy Use for Hormone Receptor–Positive, ERBB2-Negative Breast Cancer After RxPONDER Trial
Source: JAMA Netw Open. Author manuscript; Available in PMC 2026 Feb 4. (PMC12728658; doi:10.1001/jamanetworkopen.2025.49109)
Supplement: Supplement 1 — eFigure 1. CONSORT Flow Diagram for Cohort Selection of Women Diagnosed With Stage I-III, HR-Positive/HER2-Negative Breast Cancer From the National Cancer Database eTable 1. Sociodemographic and Clinicopathologic Characteristics of Women With Stage I-III, HR-Positive/HER2Negative Breast Cancer, by Genomic Risk eFigure 2. Trends in Chemotherapy Use Among Premenopausal (Ages ≤50) Women With Stage I-III, HR-Positive/HER2-Negative, Node-Negative Breast Cancer Across Genomic Risk Categories eFigure 3. Trends in Chemotherapy Use Among Premenopausal (Ages ≤50) Women With Stage I-III, HR-Positive/HER2-Negative, Node-Positive Breast Cancer Across Genomic Risk Categories eFigure 4. Trends in Chemotherapy Use Among Postmenopausal (Ages >50) Women With Stage I-III, HR-Positive/HER2-Negative, Node-Negative Breast Cancer Across Genomic Risk Categories eFigure 5. Trends in Chemotherapy Use Among Postmenopausal (Ages >50) Women With Stage I-III, HR-Positive/HER2-Negative, Node-Positive Breast Cancer Across Genomic Risk Categories eTable 2. Sociodemographic and Clinicopathologic Characteristics of Women With Stage I-III, HR-Positive/HER2-Negative Breast Cancer, by Treatment Modality eTable 3. Percentages of Adjuvant Chemotherapy Use in Women With Stage I-III, HR− Positive/HER2-Negative Breast Cancer Across Genomic Risk Categories, Overall and Stratified by Menopausal and Nodal Status eTable 4. Factors Associated with Chemoendocrine Therapy Use (vs Endocrine Therapy Alone) in Stage I-III, Hormone Receptor-Positive/HER2-Negative Breast Cancer Across Patient Cohorts, 2018–2022 [file NIHMS2132632-supplement-Supplement_1.pdf]

Supplemental Online Content

Freeman JQ, Saha P, Peiffer DS, et al. Adjuvant chemotherapy use for hormone receptor–positive, *ERBB2*-negative breast cancer after RxPONDER trial. *JAMA Netw Open*. 2025;8(12):e2549109. doi:10.1001/jamanetworkopen.2025.49109

eFigure 1. CONSORT Flow Diagram for Cohort Selection of Women Diagnosed With Stage I-III, HR-Positive/HER2-Negative Breast Cancer From the National Cancer Database

eTable 1. Sociodemographic and Clinicopathologic Characteristics of Women With Stage I-III, HR-Positive/HER2-Negative Breast Cancer, by Genomic Risk

eFigure 2. Trends in Chemotherapy Use Among Premenopausal (Ages ≤50) Women With Stage I-III, HR-Positive/HER2-Negative, Node-Negative Breast Cancer Across Genomic Risk Categories

eFigure 3. Trends in Chemotherapy Use Among Premenopausal (Ages ≤50) Women With Stage I-III, HR-Positive/HER2-Negative, Node-Positive Breast Cancer Across Genomic Risk Categories

eFigure 4. Trends in Chemotherapy Use Among Postmenopausal (Ages >50) Women With Stage I-III, HR-Positive/HER2-Negative, Node-Negative Breast Cancer Across Genomic Risk Categories

eFigure 5. Trends in Chemotherapy Use Among Postmenopausal (Ages >50) Women With Stage I-III, HR-Positive/HER2-Negative, Node-Positive Breast Cancer Across Genomic Risk Categories

eTable 2. Sociodemographic and Clinicopathologic Characteristics of Women With Stage I-III, HR-Positive/HER2-Negative Breast Cancer, by Treatment Modality

eTable 3. Percentages of Adjuvant Chemotherapy Use in Women With Stage I-III, HR-Positive/HER2-Negative Breast Cancer Across Genomic Risk Categories, Overall and Stratified by Menopausal and Nodal Status

eTable 4. Factors Associated with Chemoendocrine Therapy Use (vs Endocrine Therapy Alone) in Stage I-III, Hormone Receptor-Positive/HER2-Negative Breast Cancer Across Patient Cohorts, 2018-2022

This supplemental material has been provided by the authors to give readers additional information about their work.

© 2025 Freeman JQ et al. *JAMA Network Open*

eFigure 1. CONSORT Flow Diagram for Cohort Selection of Women Diagnosed With Stage I-III, HR-Positive/HER2-Negative Breast Cancer from the National Cancer Database

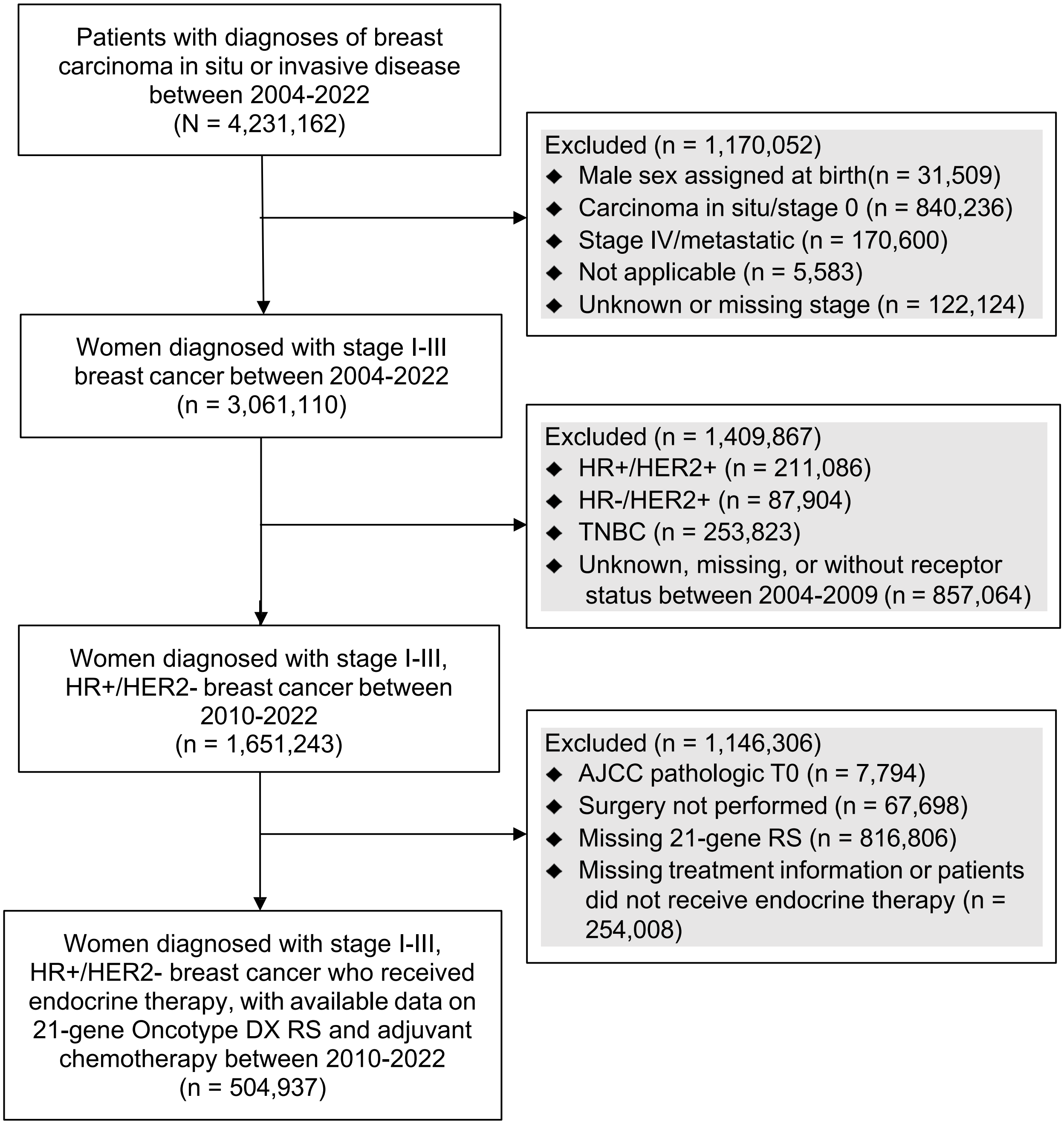

© 2025 Freeman JQ et al. *JAMA Network Open*

eTable 1. Sociodemographic and Clinicopathologic Characteristics of Women With Stage I-III, HR-Positive/HER2-Negative Breast Cancer, by Genomic Risk

	**21-gene recurrence score**			
	**Low risk (0-10)**	**Intermediate risk (11-25)**	**High risk (26-100)**	
**Characteristic**	**n = 124312 (24.6)**	**n = 308070 (61.0)**	**n = 72555 (14.4)**	
	**No. (%)**	**No. (%)**	**No. (%)**	***P* value [a]**
**Age at diagnosis**				
Mean (SD)	61.5 (10.3)	59.5 (10.7)	59.7 (11.3)	<.001
Median (IQR)	63.0 (54.0, 69.0)	60.0 (51.0, 68.0)	61.0 (52.0, 68.0)	<.001
**Stage of menopause**				
Premenopausal (ages ≤50)	21580 (17.4)	70417 (22.9)	15209 (21.0)	<.001
Postmenopausal (ages >50)	102732 (82.6)	237653 (77.1)	57346 (79.0)	
**Race/ethnicity [b]**				
White	101030 (81.9)	250133 (81.8)	56046 (77.8)	<.001
Black	8934 (7.2)	23633 (7.7)	8036 (11.2)	
Asian or Pacific Islander	5553 (4.5)	12950 (4.2)	3269 (4.5)	
Hispanic	6827 (5.5)	16273 (5.3)	3989 (5.5)	
Other	1057 (0.9)	2754 (0.9)	666 (0.9)	
**Percent no high school degree quartiles [c]**				
≥ 15.3	15897 (15.1)	37646 (14.4)	9966 (16.2)	<.001
9.1-15.2	26656 (25.4)	64532 (24.7)	15873 (25.8)	
5.0-9.0	32730 (31.2)	81734 (31.3)	18719 (30.5)	
< 5.0	29721 (28.3)	77440 (29.6)	16888 (27.5)	
**Median household income quartiles [d]**				
< $46,277	12370 (11.8)	29196 (11.2)	7821 (12.8)	<.001
$46,227-$57,856	20293 (19.4)	47916 (18.4)	12037 (19.6)	
$57,857-$74,062	25019 (23.9)	61544 (23.6)	14176 (23.1)	
≥ $74,063	47065 (44.9)	122120 (46.8)	27266 (44.5)	
**Primary payor at diagnosis**				
Uninsured	1273 (1.0)	3333 (1.1)	866 (1.2)	<.001
Private	64620 (52.4)	178234 (58.4)	40704 (56.6)	
Medicaid	6327 (5.1)	16738 (5.5)	4373 (6.1)	
Medicare	49749 (40.4)	103550 (33.9)	25091 (34.9)	

© 2025 Freeman JQ et al. *JAMA Network Open*

Other governmental	1299 (1.1)	3449 (1.1)	836 (1.2)	
**Rural-urban residence [e]**				
Metropolitan	101048 (84.1)	252300 (84.8)	59237 (84.5)	<.001
Urban	16666 (13.9)	39412 (13.2)	9484 (13.5)	
Rural	2481 (2.1)	5787 (1.9)	1385 (2.0)	
**Facility type/cancer program**				
Community	8467 (6.9)	19078 (6.4)	4671 (6.8)	<.001
Comprehensive community	50053 (40.9)	121094 (40.5)	27616 (40.0)	
Academic/research	37637 (30.8)	95876 (32.0)	22551 (32.6)	
Integrated network	26207 (21.4)	63145 (21.1)	14282 (20.7)	
**Charlson-Deyo comorbidity index**				
0	102485 (82.4)	258671 (84.0)	59787 (82.4)	<.001
1	16212 (13.0)	37450 (12.2)	9455 (13.0)	
≥2	5615 (4.5)	11949 (3.9)	3313 (4.6)	
**AJCC stage group**				
I	103672 (83.4)	248294 (80.6)	49229 (67.9)	<.001
II	19623 (15.8)	56720 (18.4)	22142 (30.5)	
III	1017 (0.8)	3056 (1.0)	1184 (1.6)	
**Histologic type**				
Ductal	97030 (78.1)	227726 (73.9)	62618 (86.3)	<.001
Lobular	14434 (11.6)	50876 (16.5)	5519 (7.6)	
Ductal and lobular	7316 (5.9)	21307 (6.9)	2877 (4.0)	
Other	5532 (4.5)	8161 (2.6)	1541 (2.1)	
**Progesterone receptor status**				
Negative	1917 (1.5)	24900 (8.1)	20906 (28.9)	<.001
Positive	122330 (98.5)	282977 (91.9)	51551 (71.1)	
**AJCC pathologic T stage**				
pT1	92712 (75.5)	224101 (73.8)	43546 (62.5)	<.001
pT2	27482 (22.4)	72728 (24.0)	24756 (35.5)	
pT3	2468 (2.0)	6309 (2.1)	1241 (1.8)	
pT4	137 (0.1)	399 (0.1)	125 (0.2)	
**AJCC pathologic nodal status**				
pN0	97836 (81.5)	241850 (81.2)	55928 (81.9)	<.001
pN1	21434 (17.9)	53761 (18.0)	11734 (17.2)	
pN2	617 (0.5)	1736 (0.6)	479 (0.7)	

© 2025 Freeman JQ et al. *JAMA Network Open*

pN3	159 (0.1)	506 (0.2)	144 (0.2)	
Negative (pN0)	97836 (81.5)	241850 (81.2)	55928 (81.9)	<.001
Positive (pN1+)	22210 (18.5)	56003 (18.8)	12357 (18.1)	
**Tumor grade**				
1	45925 (39.2)	97310 (33.2)	6455 (9.3)	<.001
2	66050 (56.3)	169442 (57.7)	33227 (47.9)	
3	5271 (4.5)	26775 (9.1)	29684 (42.8)	

Abbreviations: HR, hormone receptor; HER2, human epidermal growth factor receptor 2; No., number; SD, standard deviation; IQR, interquartile range; AJCC, American Joint Committee on Cancer.

[a] *P* values were computed using ANOVA, Kruskal-Wallis, or Pearson's chi-squared tests.

[b] Other includes American Indian, Alaska Native, Other, or unknown races/ethnicities.

[c] Defined as education attainment for patient residence areas and measured by matching the zip code of the patient recorded at the time of diagnosis against files derived from the 2020 American Community Survey data.

[d] Based on the 2020 American Community Survey data, spanning years 2016–2020 and adjusted for 2016 inflation.

[e] Measured by matching the state and county FIPS code of the patient recorded at the time of diagnosis against 2013 files published by the United States Department of Agriculture Economic Research Service.

© 2025 Freeman JQ et al. *JAMA Network Open*

eFigure 2. Trends in Chemotherapy Use among Premenopausal (ages ≤50) Women With Stage I-III, HR-Positive/HER2-Negative, Node-Negative Breast Cancer across Genomic Risk Categories

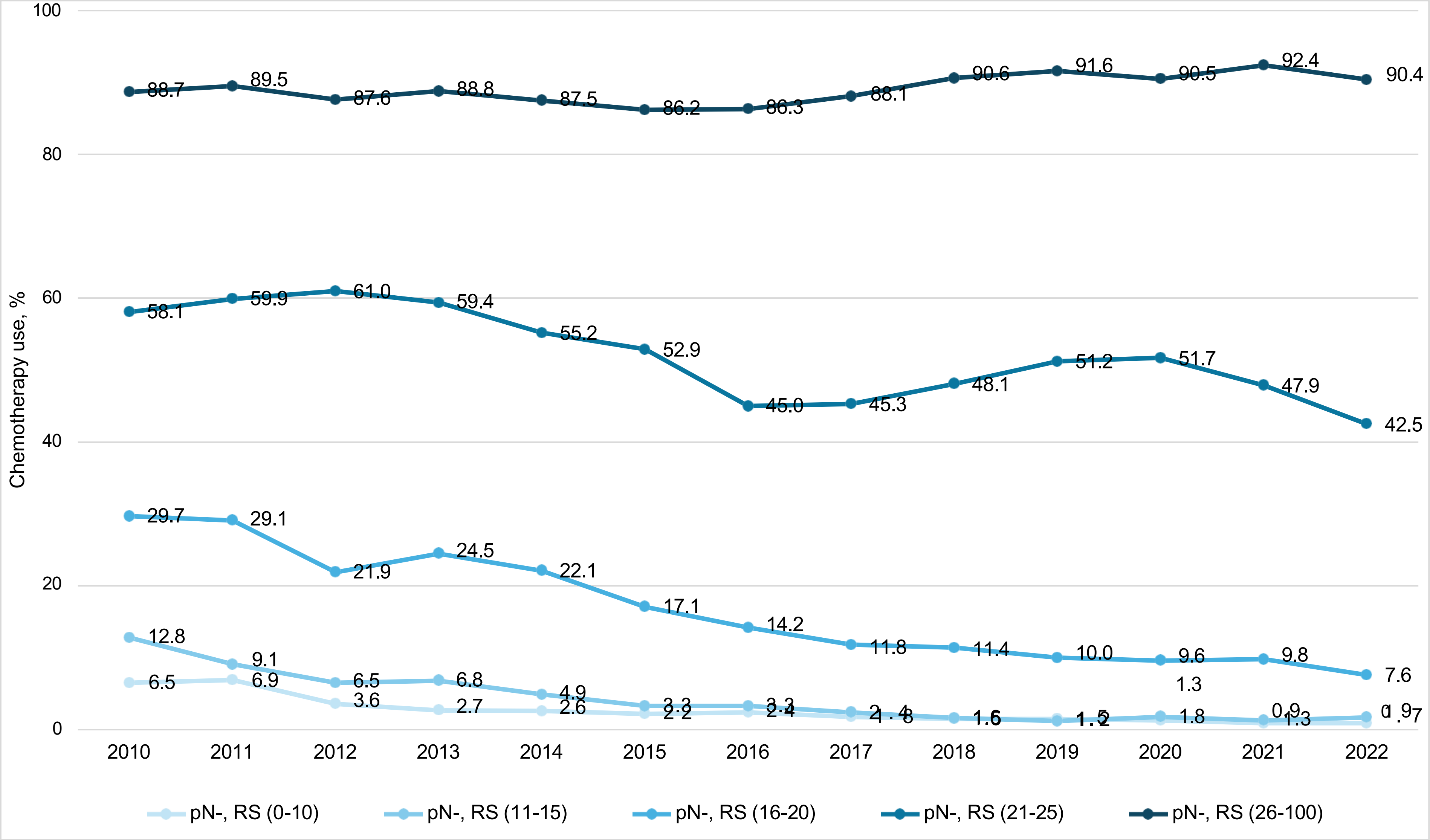

© 2025 Freeman JQ et al. *JAMA Network Open*

eFigure 3. Trends in Chemotherapy Use among Premenopausal (ages ≤50) Women With Stage I-III, HR-Positive/HER2-Negative, Node-Positive Breast Cancer across Genomic Risk Categories

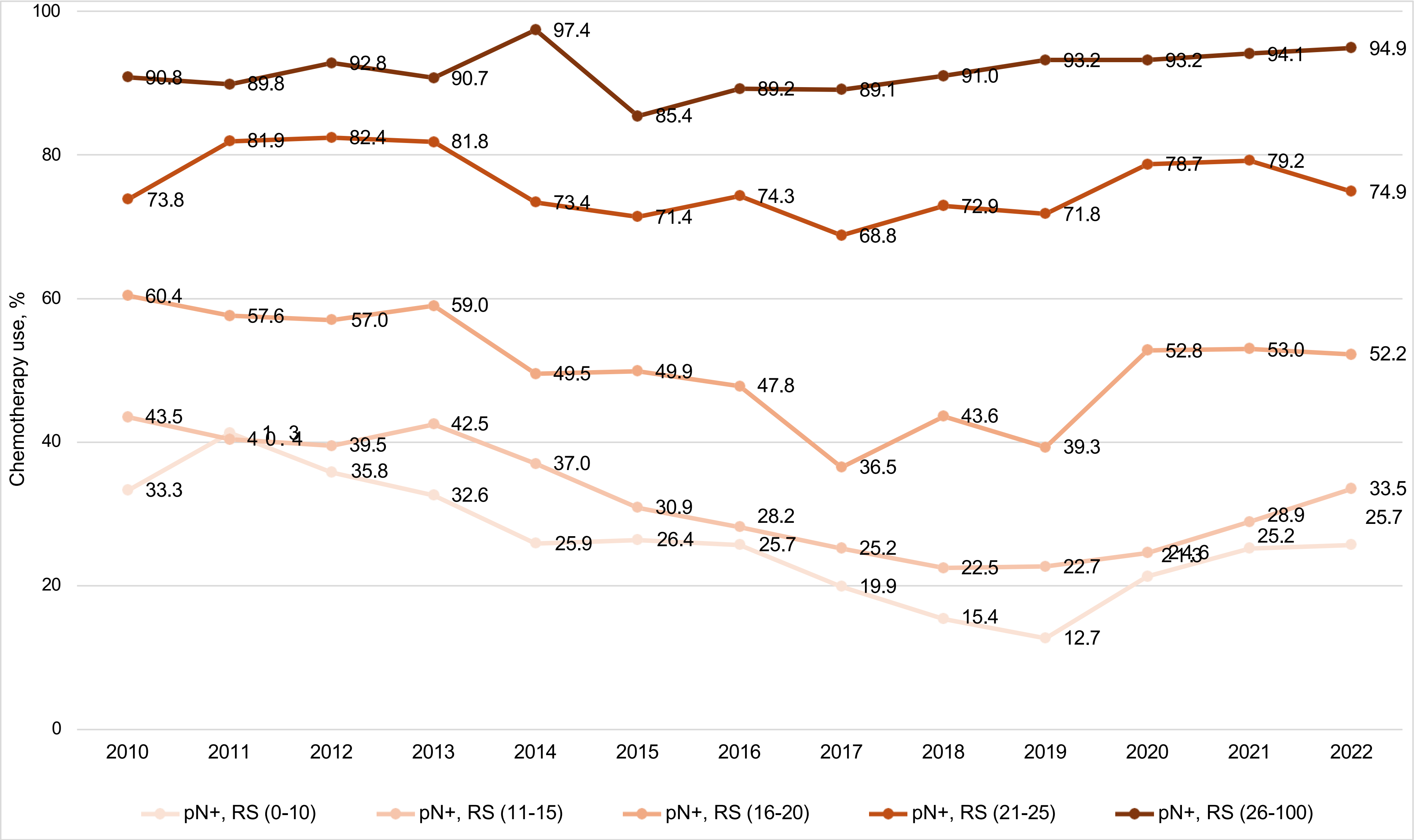

© 2025 Freeman JQ et al. *JAMA Network Open*

eFigure 4. Trends in Chemotherapy Use among Postmenopausal (ages >50) Women With Stage I-III, HR-Positive/HER2-Negative, Node-Negative Breast Cancer across Genomic Risk Categories

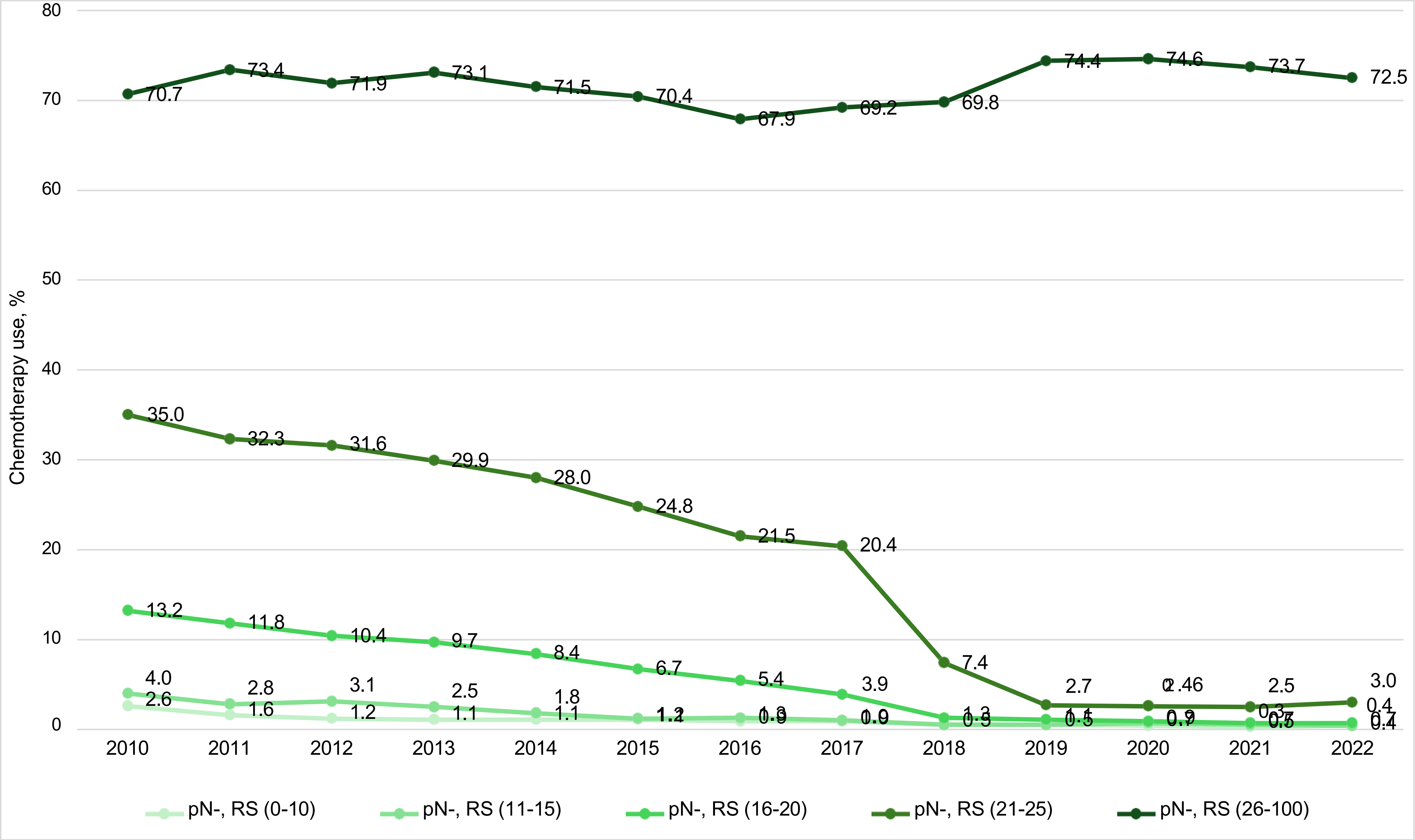

© 2025 Freeman JQ et al. *JAMA Network Open*

eFigure 5. Trends in Chemotherapy Use among Postmenopausal (ages >50) Women With Stage I-III, HR-Positive/HER2-Negative, Node-Positive Breast Cancer across Genomic Risk Categories

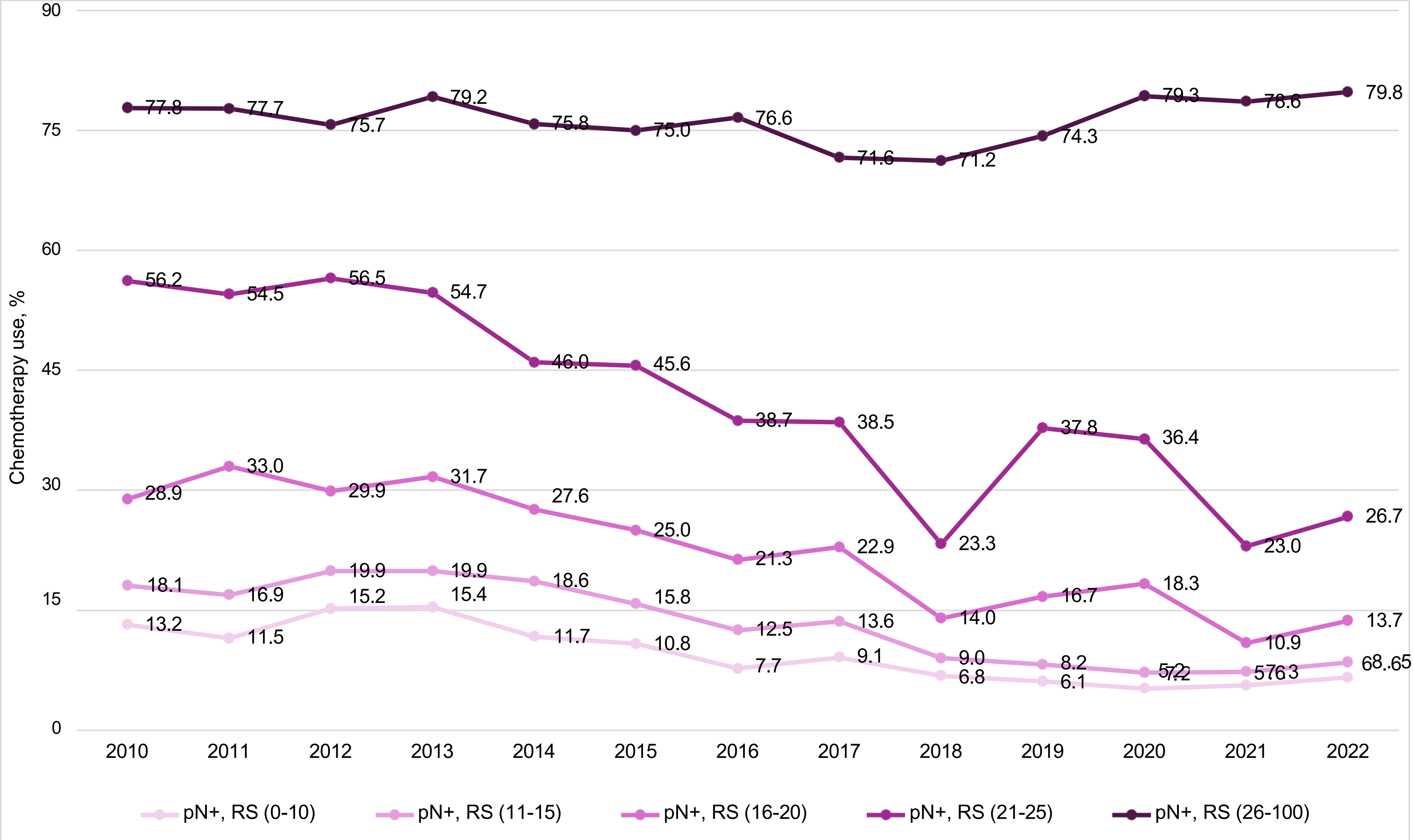

© 2025 Freeman JQ et al. *JAMA Network Open*

eTable 2. Sociodemographic and Clinicopathologic Characteristics of Women With Stage I-III, HR-Positive/HER2-Negative Breast Cancer, by Treatment Modality

Characteristic	Endocrine therapy alone<br>n = 408930 (81.2)<br>No. (%)	Chemoendocrine Therapy<br>n = 94651 (18.8)<br>No. (%)	*P* value [a]
**Age at diagnosis**			
Mean (SD)	60.9 (10.4)	56.0 (11.1)	<.001
Median (IQR)	62.0 (53.0, 69.0)	56.0 (48.0, 64.0)	<.001
**Stage of menopause**			
Premenopausal (ages ≤50)	75837 (18.5)	31081 (32.8)	<.001
Postmenopausal (ages >50)	333093 (81.5)	63570 (67.2)	
**Race/ethnicity [b]**			
White	332999 (82.0)	73198 (77.9)	<.001
Black	31075 (7.7)	9404 (10.0)	
Asian or Pacific Islander	17120 (4.2)	4575 (4.9)	
Hispanic	21133 (5.2)	5848 (6.2)	
Other	3559 (0.9)	895 (1.0)	
**Percent no high school degree quartiles [c]**			
≥ 15.3	50621 (14.6)	12662 (15.7)	<.001
9.1-15.2	86718 (25.1)	20089 (24.9)	
5.0-9.0	108170 (31.3)	24679 (30.6)	
< 5.0	100461 (29.0)	23262 (28.8)	
**Median household income quartiles [d]**			
< $46,277	39796 (11.5)	9460 (11.7)	<.001
$46,227-$57,856	64969 (18.8)	15088 (18.7)	
$57,857-$74,062	82189 (23.8)	18306 (22.7)	
≥ $74,063	158223 (45.8)	37659 (46.8)	
**Primary payor at diagnosis**			
Uninsured	4130 (1.0)	1312 (1.4)	<.001
Private	220715 (54.5)	62106 (66.2)	
Medicaid	20883 (5.2)	6484 (6.9)	
Medicare	155232 (38.3)	22679 (24.2)	
Other governmental	4374 (1.1)	1193 (1.3)	
**Rural-urban residence [e]**			
Metropolitan	333918 (84.5)	77534 (85.1)	<.001
Urban	53544 (13.5)	11867 (13.0)	
Rural	7901 (2.0)	1728 (1.9)	
**Facility type/cancer program**			
Community	26435 (6.6)	5677 (6.5)	<.001
Comprehensive community	164013 (40.9)	34180 (38.9)	
Academic/research	125849 (31.3)	29775 (33.9)	
Integrated network	85140 (21.2)	18288 (20.8)	
**Charlson-Deyo comorbidity index**			
0	339489 (83.0)	80288 (84.8)	<.001
1	51786 (12.7)	11180 (11.8)	
≥2	17655 (4.3)	3183 (3.4)	

© 2025 Freeman JQ et al. *JAMA Network Open*

**AJCC stage group**			
I	341510 (83.5)	58740 (62.1)	<.001
II	65248 (16.0)	32849 (34.7)	
III	2172 (0.5)	3062 (3.2)	
**Histologic type**			
Ductal	308062 (75.3)	78287 (82.7)	<.001
Lobular	61071 (14.9)	9555 (10.1)	
Ductal and lobular	26589 (6.5)	4828 (5.1)	
Other	13208 (3.2)	1981 (2.1)	
**Progesterone receptor status**			
Negative	28236 (6.9)	19356 (20.5)	<.001
Positive	380438 (93.1)	75196 (79.5)	
**AJCC pathologic T stage**			
pT1	305744 (75.6)	53755 (59.6)	<.001
pT2	91417 (22.6)	33151 (36.7)	
pT3	6847 (1.7)	3127 (3.5)	
pT4	429 (0.1)	230 (0.3)	
**AJCC pathologic nodal status**			
pN0	332308 (84.0)	62356 (69.8)	<.001
pN1	62349 (15.8)	24268 (27.2)	
pN2	812 (0.2)	2003 (2.2)	
pN3	154 (<1)	651 (0.7)	
Negative (pN0)	332308 (84.0)	62356 (69.8)	<.001
Positive (pN1+)	63315 (16.0)	26922 (30.2)	
**Tumor grade**			
1	137693 (35.5)	11660 (12.9)	<.001
2	220657 (56.8)	47283 (52.2)	
3	29952 (7.7)	31611 (34.9)	

Abbreviations: HR, hormone receptor; HER2, human epidermal growth factor receptor 2; No., number; SD, standard deviation; IQR, interquartile range; AJCC, American Joint Committee on Cancer.

[a] *P* values were computed using *t*-tests, Wilcoxon rank-sum, or Pearson's chi-squared tests.

[d] Other includes American Indian, Alaska Native, Other, or unknown races/ethnicities.

[c] Defined as education attainment for patient residence areas and measured by matching the zip code of the patient recorded at the time of diagnosis against files derived from the 2020 American Community Survey data.

[d] Based on the 2020 American Community Survey data, spanning years 2016–2020 and adjusted for 2016 inflation.

[e] Measured by matching the state and county FIPS code of the patient recorded at the time of diagnosis against 2013 files published by the United States Department of Agriculture Economic Research Service.

© 2025 Freeman JQ et al. *JAMA Network Open*

eTable 3. Percentages of Adjuvant Chemotherapy Use in Women With Stage I-III, HR-Positive/HER2-Negative Breast Cancer across Genomic Risk Categories, Overall and Stratified by Menopausal and Nodal Status

		**Endocrine therapy alone**	**Chemoendocrine Therapy**	
		**n = 408930 (81.2)**	**n = 94651 (18.8)**	
**Characteristic**	**21-gene RS**	**No. (row %)**	**No. (row %)**	***P* value [a]**
**All women**				
	Low risk (0-10)	120258 (97.0)	3737 (3.0)	<.001
	Intermediate risk (11-25)	271608 (88.4)	35582 (11.6)	
	High risk (26-100)	17064 (23.6)	55332 (76.4)	
**AJCC pathologic nodal status**				
Negative (pN0)				
	Low risk (0-10)	96630 (99.0)	983 (1.0)	<.001
	Intermediate risk (11-25)	221977 (92.0)	19264 (8.0)	
	High risk (26-100)	13701 (24.5)	42109 (75.5)	
Positive (pN1+)				
	Low risk (0-10)	19672 (88.9)	2462 (11.1)	<.001
	Intermediate risk (11-25)	41169 (73.8)	14603 (26.2)	
	High risk (26-100)	2474 (20.1)	9857 (79.9)	
pN1				
	Low risk (0-10)	19384 (90.7)	1975 (9.3)	<.001
	Intermediate risk (11-25)	40575 (75.8)	12971 (24.2)	
	High risk (26-100)	2390 (20.4)	9322 (79.6)	
pN2-3				
	Low risk (0-10)	288 (37.2)	487 (62.8)	<.001
	Intermediate risk (11-25)	594 (26.7)	1632 (73.3)	
	High risk (26-100)	84 (13.6)	535 (86.4)	
**Premenopausal (ages ≤50) women**				
**Overall**				
	Low risk (0-10)	20166 (93.7)	1361 (6.3)	<.001
	Intermediate risk (11-25)	54190 (77.2)	16013 (22.8)	
	High risk (26-100)	1481 (9.8)	13707 (90.2)	
**AJCC pathologic nodal status**				
Negative (pN0)				

© 2025 Freeman JQ et al. *JAMA Network Open*

	Low risk (0-10)	17027 (97.8)	375 (2.2)	<.001
	Intermediate risk (11-25)	46542 (83.2)	9381 (16.8)	
	High risk (26-100)	1233 (10.7)	10257 (89.3)	
Positive (pN1+)				
	Low risk (0-10)	2905 (76.0)	920 (24.0)	<.001
	Intermediate risk (11-25)	6957 (53.9)	5957 (46.1)	
	High risk (26-100)	221 (9.0)	2556 (92.0)	
pN1				
	Low risk (0-10)	2885 (78.3)	798 (21.7)	<.001
	Intermediate risk (11-25)	6895 (55.6)	5516 (44.4)	
	High risk (26-100)	216 (8.2)	2407 (91.8)	
pN2-3				
	Low risk (0-10)	20 (14.1)	122 (85.9)	.003
	Intermediate risk (11-25)	62 (12.3)	441 (87.7)	
	High risk (26-100)	5 (3.3)	149 (96.8)	
**Postmenopausal (ages >50) women**				
**Overall**				
	Low risk (0-10)	100092 (97.7)	2376 (2.3)	<.001
	Intermediate risk (11-25)	217418 (91.7)	19569 (8.3)	
	High risk (26-100)	15583 (27.2)	41625 (72.8)	
**AJCC pathologic nodal status**				
Negative (pN0)				
	Low risk (0-10)	79603 (99.2)	608 (0.8)	<.001
	Intermediate risk (11-25)	175435 (94.7)	9883 (5.3)	
	High risk (26-100)	12468 (28.1)	31852 (71.9)	
Positive (pN1+)				
	Low risk (0-10)	16767 (91.6)	1542 (8.4)	<.001
	Intermediate risk (11-25)	34212 (79.8)	8646 (20.2)	
	High risk (26-100)	2253 (23.6)	7301 (76.4)	
pN1				
	Low risk (0-10)	16499 (93.3)	1177 (6.7)	<.001
	Intermediate risk (11-25)	33680 (81.9)	7455 (18.1)	
	High risk (26-100)	2174 (23.9)	6915 (76.1)	
pN2-3				
	Low risk (0-10)	268 (42.3)	365 (57.7)	<.001

© 2025 Freeman JQ et al. *JAMA Network Open*

	Intermediate risk (11-25)	532 (30.9)	1191 (69.1)	
	High risk (26-100)	79 (17.0)	386 (83.0)	

Abbreviations: HR, hormone receptor; HER2, human epidermal growth factor receptor 2; AJCC, American Joint Committee on Cancer; No., number; RS, recurrence score.

[a] *P* values were calculated using Pearson's chi-squared tests.

© 2025 Freeman JQ et al. *JAMA Network Open*

eTable 4. Factors Associated with Chemoendocrine Therapy Use (vs Endocrine Therapy Alone) in Stage I-III, Hormone Receptor-Positive/HER2-Negative Breast Cancer across Patient Cohorts, 2018-2022

	**Cohort 1:** women with genomic high risk (RS ≥26), regardless of menopausal and nodal status	**Cohort 2:** premenopausal (ages ≤50) women with pN0 and RS <16; postmenopausal (ages >50) women with pN0/1+ and RS <26	**Cohort 3:** premenopausal (ages ≤50) women with pN0 and RS 16-25 or with pN1+ and low-intermediate risk (RS 0-25)
**Characteristic**	**n = 37220**	**n = 203220**	**n = 23627**
	**AOR (95% CI)**	**AOR (95% CI)**	**AOR (95% CI)**
**Race/ethnicity [a]**			
Black	**0.76 (0.68-0.85)**	1.02 (0.90-1.15)	**0.83 (0.71-0.96)**
Asian or Pacific Islander	0.95 (0.82-1.11)	0.96 (0.82-1.13)	0.93 (0.80-1.08)
Hispanic	1.02 (0.88-1.19)	1.12 (0.97-1.28)	1.14 (0.99-1.32)
Other	0.77 (0.57-1.05)	0.94 (0.68-1.29)	0.84 (0.58-1.23)
White	1 [Reference]	1 [Reference]	1 [Reference]
**Age (year) group**			
≤40	1 [Reference]	1 [Reference]	1 [Reference]
41-50	**0.60 (0.36-1.00)**	0.64 (0.36-1.14)	**0.60 (0.51-0.70)**
51-60	**0.36 (0.21-0.59)**	**0.51 (0.29-0.87)**	NA
61-70	**0.25 (0.15-0.42)**	**0.33 (0.28-0.58)**	NA
≥71	**0.10 (0.06-0.17)**	**0.18 (0.10-0.31)**	NA
**AJCC pathologic nodal status**			
Negative (pN0)	1 [Reference]	1 [Reference]	1 [Reference]
Positive (pN1+)	**1.352(1.21-1.43)**	**11.69 (10.85-12.59)**	**6.45 (5.85-7.13)**
**21-gene RS**	**1.05 (1.05-1.06)**	**1.14 (1.13-1.14)**	**1.27 (1.26-1.28)**
**AJCC pathologic T stage**			
pT1	1 [Reference]	1 [Reference]	1 [Reference]
pT2	**1.10 (1.02-1.17)**	**1.83 (1.70-1.97)**	**1.65 (1.51-1.80)**
pT3	0.89 (0.72-1.11)	**6.33 (5.59-7.17)**	**5.17 (4.17-6.42)**
pT4	1.09 (0.52-2.29)	**10.48 (6.83-16.09)**	2.04 (0.26-16.03)
**Histologic type**			
Ductal	1 [Reference]	1 [Reference]	1 [Reference]
Lobular	0.90 (0.81-1.00)	0.96 (0.88-1.05)	**0.79 (0.69-0.89)**
Ductal and lobular	0.91 (0.78-1.07)	1.05 (0.92-1.20)	0.87 (0.73-1.04)

© 2025 Freeman JQ et al. *JAMA Network Open*

Other	1.02 (0.78-1.33)	0.98 (0.74-1.30)	**0.66 (0.47-0.92)**
**Tumor grade**			
1	1 [Reference]	1 [Reference]	1 [Reference]
2	**1.57 (1.47-1.69)**	**1.42 (1.31-1.54)**	**1.43 (1.30-1.57)**
3	**2.38 (2.21-2.56)**	**2.68 (2.39-3.02)**	**2.49 (2.14-2.90)**
**Charlson-Deyo comorbidity index**			
0	1 [Reference]	1 [Reference]	1 [Reference]
1	**1.30 (1.14-1.47)**	**1.19 (1.02-1.40)**	0.97 (0.69-1.35)
≥2	**1.36 (1.17-1.57)**	1.14 (0.96-1.37)	1.07 (0.74-1.53)
**Years of initial diagnosis**	**1.08 (1.06-1.11)**	**0.91 (0.89-0.93)**	1.03 (1.00-1.06)
**Percent no high school degree quartile [b]**			
≥ 15.3	1 [Reference]	1 [Reference]	1 [Reference]
9.1-15.2	1.01 (0.91-1.12)	0.94 (0.84-1.05)	0.89 (0.77-1.02)
5.0-9.0	1.06 (0.95-1.19)	0.94 (0.84-1.06)	0.97 (0.83-1.12)
< 5.0	1.06 (0.93-1.20)	0.93 (0.82-1.06)	0.89 (0.76-1.04)
**Median household income quartile [c]**			
< $46,277	0.95 90.84-1.07)	**0.86 (0.75-0.98)**	0.97 (0.81-1.15)
$46,227-$57,856	1.00 (0.91-1.10)	0.95 (0.86-1.05)	0.94 (0.82-1.08)
$57,857-$74,062	0.94 (0.86-1.03)	**0.90 (0.82-0.98)**	**0.89 (0.80-0.99)**
≥ $74,063	1 [Reference]	1 [Reference]	1 [Reference]
**Primary payor at diagnosis**			
Uninsured	0.92 (0.66-1.29)	0.97 (0.71-1.32)	0.90 (0.66-1.22)
Private	1 [Reference]	1 [Reference]	1 [Reference]
Medicaid	**0.76 (0.65-0.88)**	1.08 (0.95-1.24)	0.93 (0.81-1.08)
Medicare	**0.69 (0.63-0.75)**	**0.85 (0.77-0.93)**	**0.64 (0.48-0.86)**
Other governmental	0.95 (0.69-1.31)	0.80 (0.58-1.10)	0.92 (0.66-1.27)
**Facility type**			
Community	1.03 (0.90-1.07)	1.08 (0.95-1.24)	0.95 (0.80-1.14)
Comprehensive community	0.99 (0.92-1.07)	1.07 (0.99-1.16)	**0.89 (0.81-0.98)**
Academic/research	1 [Reference]	1 [Reference]	1 [Reference]
Integrated network	**1.12 (1.02-1.23)**	0.97 (0.88-1.06)	0.97 (0.87-1.09)

Abbreviations: HER2, human epidermal growth factor receptor 2; RS, recurrence score; AJCC, American Joint Committee on Cancer; AOR, adjusted odds ratio; CI, confidence interval; NA, not applicable.

[a] Other includes American Indian, Alaska Native, Other, or unknown races/ethnicities.

© 2025 Freeman JQ et al. *JAMA Network Open*

[b] Defined as education attainment for patient residence areas and measured by matching the zip code of the patient recorded at the time of diagnosis against files derived from the 2020 American Community Survey data.
[c] Based on the 2020 American Community Survey data, spanning years 2016–2020 and adjusted for 2016 inflation.

© 2025 Freeman JQ et al. *JAMA Network Open*
