# Supplementary material for: Adjuvant Chemotherapy Use for Hormone Receptor–Positive, ERBB2-Negative Breast Cancer After RxPONDER Trial
Source: JAMA Netw Open. Author manuscript; Available in PMC 2026 Feb 4. (PMC12728658; doi:10.1001/jamanetworkopen.2025.49109)
Supplement: Supplement 2 — Data Sharing Statement [file NIHMS2132632-supplement-Supplement_2.pdf]

Data Sharing Statement

Freeman. Adjuvant Chemotherapy Use for Hormone Receptor–Positive, ERBB2-Negative Breast Cancer After RxPONDER Trial. *JAMA Netw Open*. Published December 23, 2025. doi:10.1001/jamanetworkopen.2025.49109

Data
Data available: No

Additional Information
Explanation for why data not available: Data types: De-identified patient data How to access data: Data for this retrospective study were collected from patients diagnosed with breast cancer from 2010 to 2022 registered in the National Cancer Database (NCDB). Investigators associated with Commission on Cancer-accredited cancer centers can request the NCDB dataset by submitting a Participant User Data File application directly to the American College of Surgeons through https://www.facs.org/quality-programs/cancer-programs/national- cancer-database. Supporting Documents Document types: None Additional Information Who can access the data: Anyone requesting the data Types of analyses: Any purpose Mechanisms of data availability: Requires a Participant User Data File application to the American College of Surgeons and a signed data use agreement according to the National Cancer Database https://www.facs.org/quality-programs/cancer-programs/national-cancer- database/puf/.
